# 

**Published:** 1891-04-25

**Authors:** 


					AifBfe - In the*' TanceV^oMffov^^ "?
II L price with a preparation of meat, combining' in itself the alnnmir.^^? iu ^er?^ possible to famish the market at a reaaonnhln
IIV K ?s5 ssmws?1 'Ssaatsa&a
U Wenti^^ri?h whe?^ts^the hn.man system and the ' \l\ ^ ji^l /J^T^ the albumen and fibrinein the most perfect wTsibkf o^f1^0;
^caa^^jsacsir?-? *> ** ^=^*iasffJvsasaSs?SM
SOLD THROUGHOUT THE UNITED KINGDOM?
.ahd.NuRWICH hospital
.tKsoan Westers Infirm art
,, I, WITH EXTRA NURSING SUPPLEMENT,
No. 239. Vol. X.] Saturday, April 25th, 1891.

				

